# Sinonasal Glomangiopericytoma: A Report of Two Cases

**DOI:** 10.7759/cureus.94694

**Published:** 2025-10-16

**Authors:** Abdulrahman Almohammed, Mahmoud Bardisi, Mohammad Dababo, Nasser M AlMadan

**Affiliations:** 1 Pathology Department, College of Medicine, Imam Mohammad Ibn Saud Islamic University (IMSIU), Riyadh, SAU; 2 Pathology Department, Riyadh Regional Laboratory and Blood Bank, Riyadh, SAU; 3 Anatomic Pathology Department, King Faisal Specialist Hospital and Research Centre, Riyadh, SAU; 4 Dental Department, Ministry of Health, Hafar al-Batin, SAU; 5 Dental Department, Prince Sultan Military Medical City, Riyadh, SAU

**Keywords:** glomangiopericytoma, hemangiopericytoma, mesenchymal sinonasal tumor, sinonasal glomangiopericytoma, spindle cell sinonasal tumor

## Abstract

Sinonasal glomangiopericytoma (GPC) is a rare mesenchymal tumor that is seen exclusively in the sinonasal region, which shows perivascular myoid differentiation. Here, we describe two cases of sinonasal glomangiopericytoma.

The first case was a 62-year-old female patient with right-sided nasal obstruction and headache. Endoscopic examination revealed a polypoid mass in the right nostril. Surgical removal revealed spindle cell proliferation with staghorn vasculature surrounded by perivascular hyalinization beneath an intact ciliated respiratory epithelium. Tumor cells were reactive to smooth muscle actin (SMA) and had nuclear staining for beta-catenin, consistent with sinonasal glomangiopericytoma. The second case was reported in a 65-year-old female patient who complained of right ear discharge with recurrent ear infections. Imaging revealed an opacification of the left ethmoid and sphenoid sinuses that was surgically removed and showed uniform spindle cells arranged in a whorling pattern with prominent vasculature and perivascular hyalinization with positive staining with SMA and nuclear reactivity to beta-catenin.

Sinonasal glomangiopericytoma is a rare mesenchymal neoplasm that can present with various clinical features, including headache, nasal obstruction, ear infection, and ear discharge. Surgical excision is the treatment of choice with rare recurrence.

## Introduction

Sinonasal glomangiopericytoma (GPC) is a rare mesenchymal neoplasm of the sinus and paranasal sinuses that can be seen in patients of all age groups, peaking in the sixth to seventh decade with a slight female predilection. It has an indolent behavior with a recurrence rate of around 20% mostly due to incomplete removal [1]. Pathologically, it shows a well-defined, unencapsulated mass in the submucosa with a tumor-free zone separating it from the intact mucosal lining. Tumor cells are spindle to ovoid arranged in a syncytial arrangement with prominent staghorn blood vessels that have peripheral hyalinization [1]. Tumor cells are reactive to smooth muscle actin (SMA), beta-catenin, lymphoid enhancer-binding factor 1 (LEF1), and cyclin D1, and it is associated with catenin beta-1 (*CTNNB1*) mutation [1]. Differential diagnosis includes solitary fibrous tumor, biphenotypic sinonasal sarcoma, and synovial sarcoma [1]. We herein report two cases of sinonasal glomangiopericytoma diagnosed at our institute.

## Case presentation

Case 1

**Figure 1:**
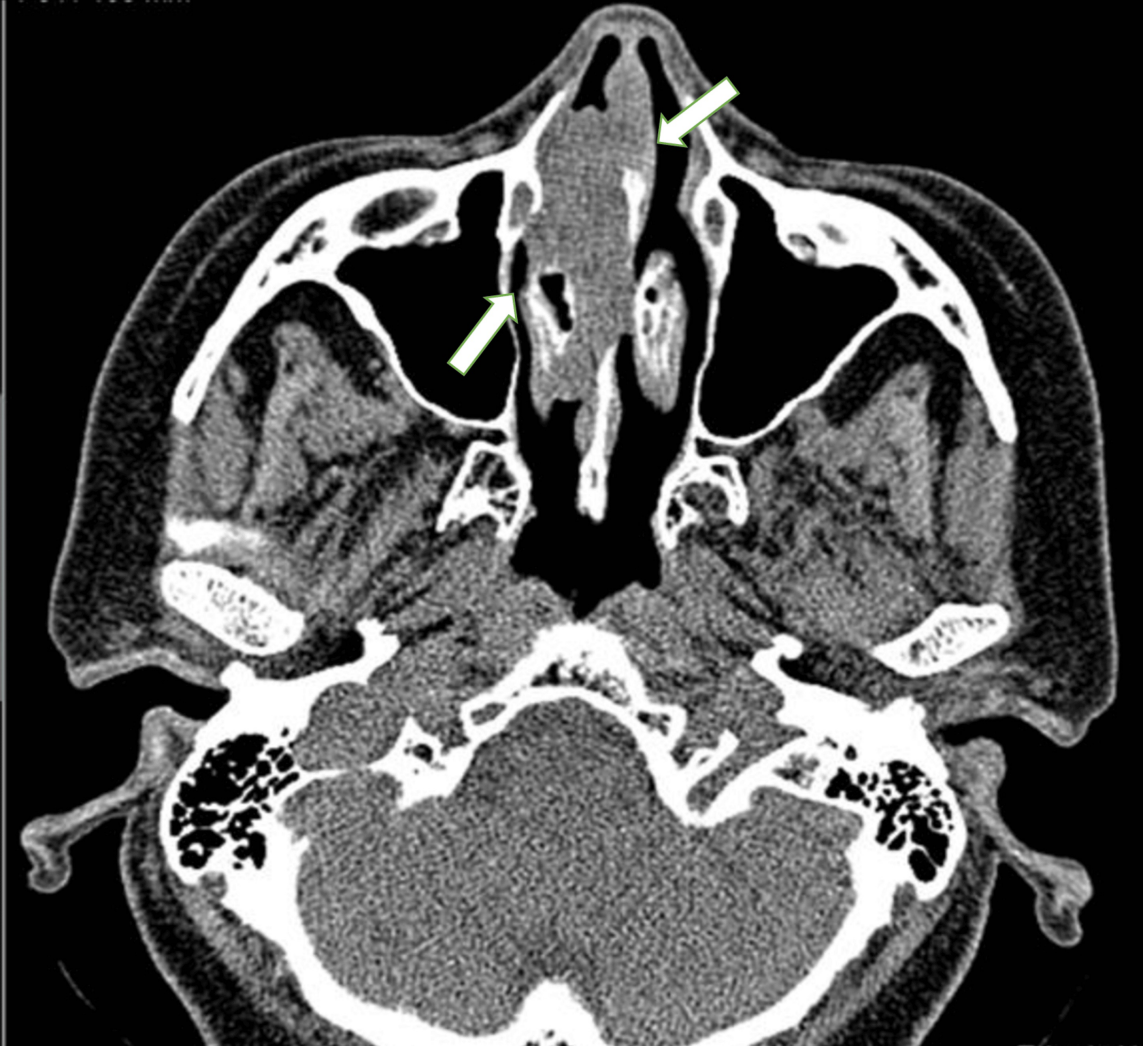
Plain CT of the nose and paranasal sinus: axial view showing an isodense mass in the right nasal cavity with erosion of the nasal septum. CT: computed tomography

A 62-year-old woman, medically healthy, was referred to King Faisal Specialist Hospital and Research Centre, Riyadh, Saudi Arabia, complaining of right nasal obstruction for four months associated with headache and facial pain; endoscopic examination revealed a polypoid mass visible through the right nostril. Computed tomography (CT) scan showed a mass occupying the right nasal cavity (Figure 1).

**Figure 2:**
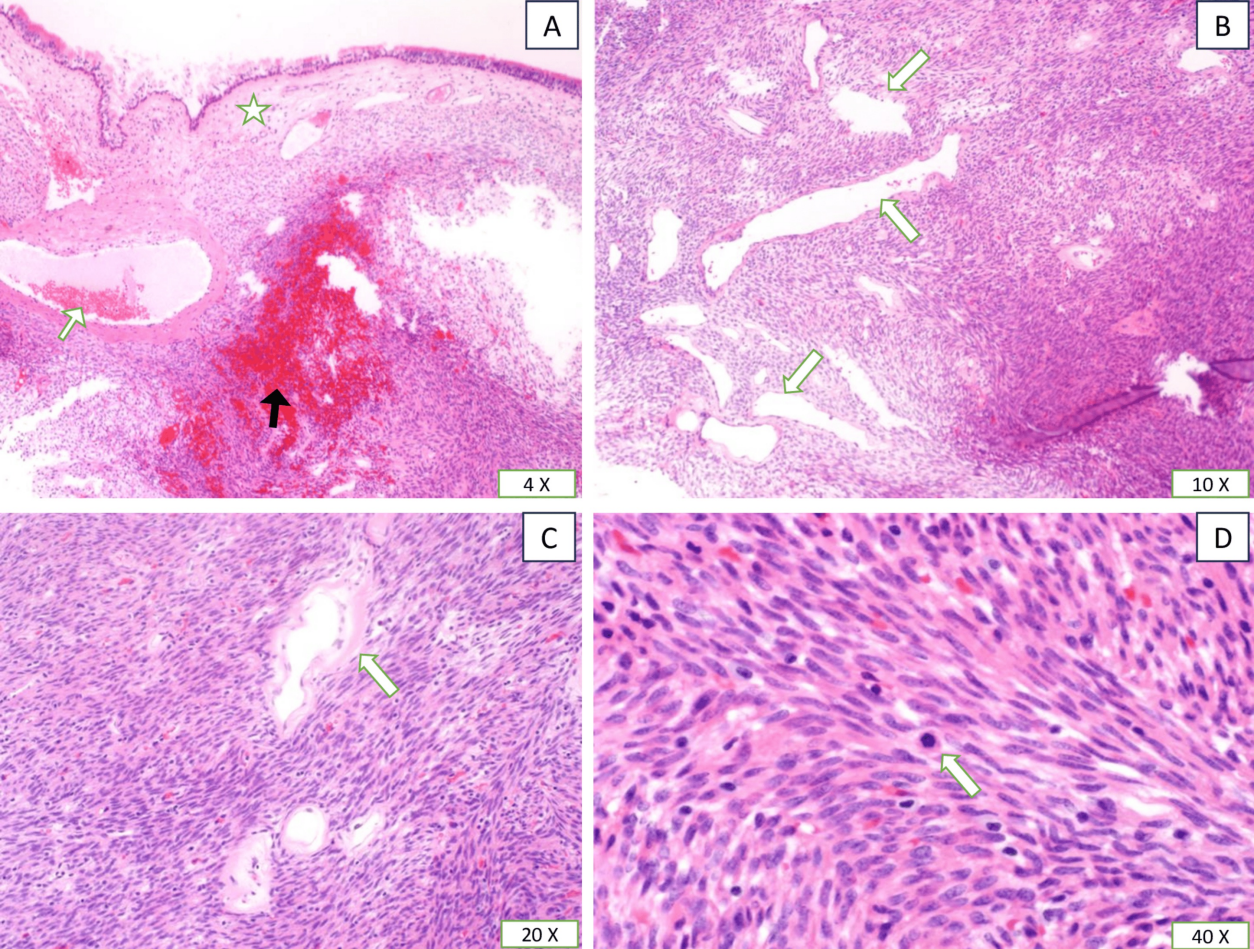
(A) Unencapsulated but well-delineated submucosal mass with hemorrhage (black arrow), prominent staghorn blood vessels (white arrow), and grenz zone separating the mass from the normal sinus mucosa (star). (B) The neoplastic cells are bland spindle cells with fusiform nuclei arranged in short fascicles with variably sized blood vessels (white arrows). (C) Higher-power view showing staghorn blood vessels with perivascular hyalinization (white arrow). (D) Mitosis was identified, but it was rare, and no atypical form was identified (white arrow).

The patient underwent endoscopic removal of the lesion, with macroscopic examination of the specimen revealing a fragmented tan, soft tissue lesion measuring 2.5 × 2.5 × 1.5 cm, while histological examination showed unencapsulated submucosal neoplastic growth with staghorn (hemangiopericytoma-like) vascular pattern beneath an intact ciliated respiratory epithelium. The neoplastic cells were bland spindle cells with fusiform nuclei and vesicular chromatin arranged in short fascicles around variable-sized vascular spaces that showed prominent perivascular hyalinization with scattered inflammatory cells, including eosinophils and mast cells. Mitotic figures were rare, while no necrosis was identified (Figure 2).

**Figure 3:**
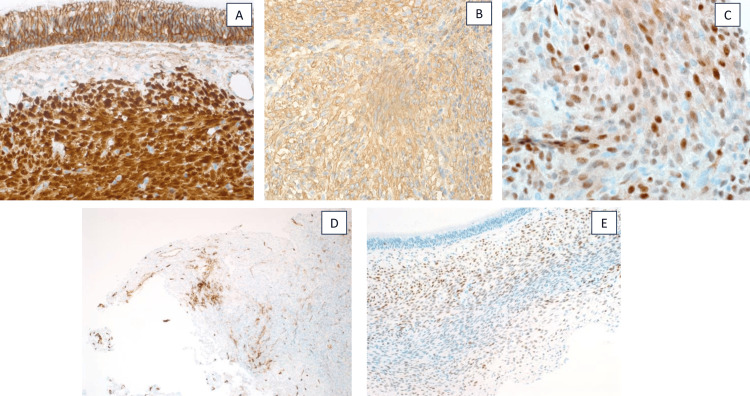
Tumor cells were reactive to beta-catenin (nuclear) (A), SMA (B), cyclin D1 (C), CD34 (focally) (D), and TLE-1 (focally) (E). SMA, smooth muscle actin; CD34, cluster of differentiation 34; TLE-1, transducin-like enhancer of split-1

Tumor cells were reactive to SMA, cluster of differentiation 34 (CD34) (focally), transducin-like enhancer of split-1 (TLE-1) (focally), and cyclin D1, with nuclear staining of beta-catenin, while it was not reactive to cytokeratins. Signal transducer and activator of transcription 6 (STAT6), desmin, and S100 are consistent with sinonasal glomangiopericytoma (Figure 3).

The patient was followed up with no sign of recurrence after 12 months.

Case 2

**Figure 4:**
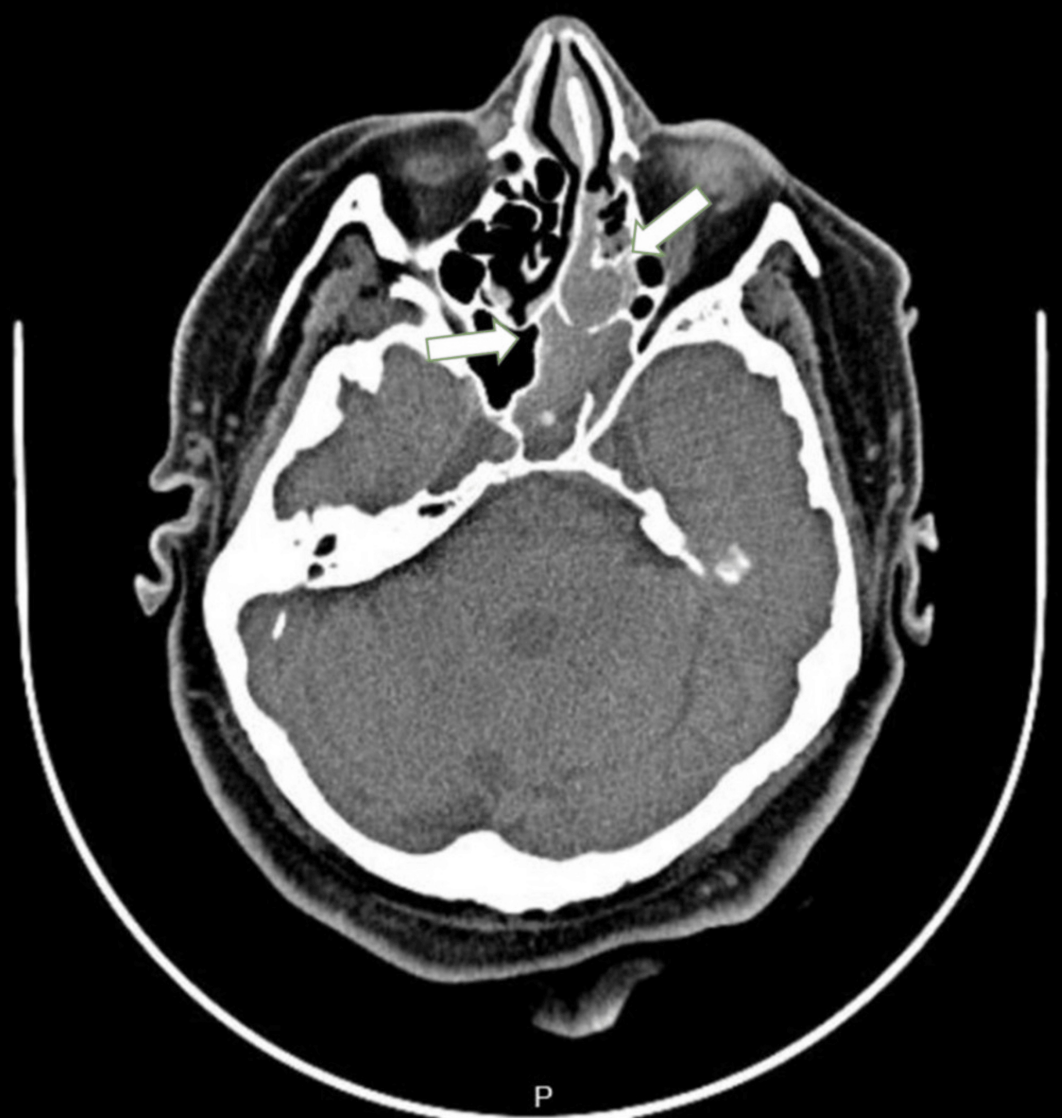
Plain CT scan showing an isodense mass in the right ethmoid sinus extending to the sphenoid sinus. CT: computed tomography

A 65-year-old woman with a known case of diabetes mellitus, hypertension, and dyslipidemia was admitted to our hospital complaining of right ear discharge with recurrent ear infections. Radiological examinations reveal the opacification of the left ethmoid and sphenoid sinuses (Figure 4).

**Figure 5:**
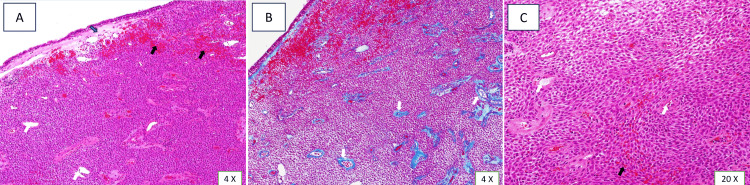
(A) Hematoxylin and eosin-stained tissue showing submucosal spindle cell proliferation with grenz zone separating the tumor from the sinonasal mass (blue arrow), hemorrhage (black arrow), and staghorn blood vessels (white arrow). (B) Masson trichrome highlighting the perivascular hyalinization (white arrows). (C) Higher power showing syncytial growth of spindle cells arranged in a whorled pattern with staghorn blood vessels (white arrows) surrounded by perivascular hyalinization and the presence of extravasated red blood cells (black arrows).

Gross specimen was a polypoid piece of tanned dusky hemorrhagic tissue measuring 2.0 × 1.5 × 1.0 cm; histologically, a polypoid structure lined by intact respiratory epithelium, the neoplastic cells were uniform with a moderate amount of cytoplasm arranged in a whorled pattern around vascular spaces predominantly showing perivascular hyalinization with rounded nuclei and vesicular chromatin (Figure 5).

**Figure 6:**
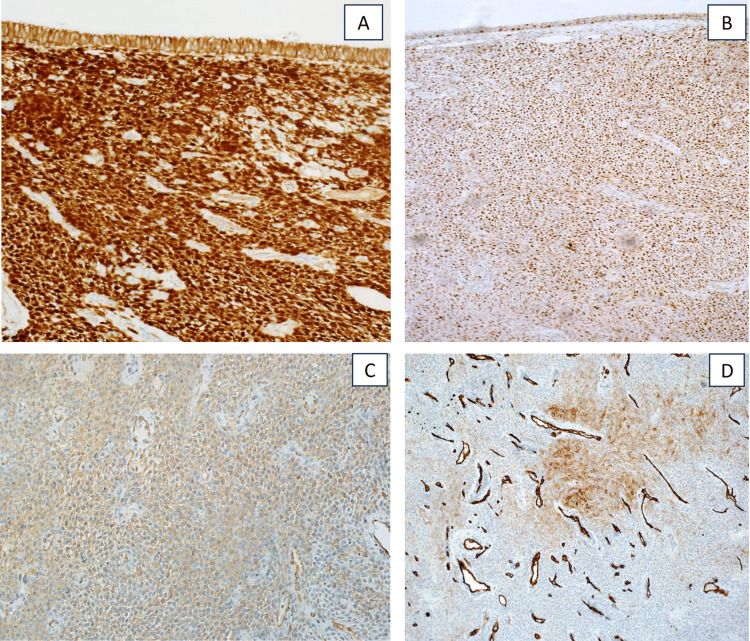
Tumor cells were reactive to beta-catenin (nuclear) (A), cyclin D1 (B), SMA (C), and CD34 (focally) (D). SMA, smooth muscle actin; CD34, cluster of differentiation 34

Immunohistochemical studies showed positive staining with beta-catenin (nuclear), cyclin D1, SMA, and CD34 (focally) while negative for cytokeratins, STAT6, desmin, and S100 (Figure 6).

The patient was followed up for three years with no sign of recurrence.

## Discussion

Glomangiopericytoma is a sinonasal tumor of mesenchymal origin with perivascular myoid differentiation [1]. Most glomangiopericytomas arise in the nasal cavity, including the turbinate and nasal septum. They often show a unilateral distribution. Usually, the tumor extends to the paranasal sinus, while isolated sinus involvement is rare [1]. It can be diagnosed from infancy to old age, though peaks occur in the sixth and seventh decades, with a slight female predilection [1]. Both our patients were women in the seventh decade, which aligns with this pattern. The main symptoms are nasal obstruction and epistaxis, with rare cases presenting oncologic osteomalacia. Álvarez-Rivas et al. systematically reviewed 1979 cases of tumor-associated osteomalacia reported until 2023, finding that 0.8% involved glomangiopericytoma [2]. Imaging shows a destructive polypoid mass with bone erosion and concurrent sinusitis [1]. MRI reveals a hyperintense signal on T2-weighted images, several vascular signal voids, and a high mean apparent diffusion coefficient (ADC) value. This indicates a low-cellularity lesion and reflects its indolent behavior. On dynamic contrast-enhanced MRI (DCE-MRI), rapid wash-in and washout patterns are seen [3].

Histologically, the lesion is well-defined yet unencapsulated and located in the submucosa with a grenz zone. Tumor cells are spindled to ovoid, with eosinophilic to clear cytoplasm and blunt-end nuclei, arranged in fascicular, whorled, or storiform patterns. The lesion features large staghorn blood vessels with perivascular hyalinization. GPC also contains extravasated red blood cells (RBCs), eosinophils, and mast cells. Limited mitotic figures may be seen, but atypical mitosis is absent. The presence of atypical mitosis, nuclear pleomorphism, and necrosis suggests aggressive behavior [1]. The immunohistochemical profile includes positive staining with SMA, cyclin D1, nuclear β-catenin, and LEF1. CD34 reactivity varies depending on the clone used. The tumor is not reactive to pancytokeratin, CD31, STAT6, S100, or SOX10 [1]. Both our cases showed beta-catenin, cyclin D1, and SMA positivity, while both were focally reactive to CD34. This can lead to a diagnosis of solitary fibrous tumor, but STAT6 can resolve this diagnostic pitfall [4]. In addition, the first case was focally reactive to TLE-1, raising the possibility of synovial sarcoma, which can also show nuclear beta-catenin reaction [5]. However, synovial sarcoma has distinct histological and immunohistochemical patterns, including negative CD34 staining, that differentiate it [6]. Another diagnostic pitfall is biphenotypic sinonasal sarcoma, characterized by unencapsulated spindle cell proliferation in long fascicles with a herringbone pattern, the entrapment of respiratory epithelium, and staghorn blood vessels [7]. It is reactive to S100 and SMA with nuclear β-catenin expression in most cases. This can cause confusion, but biphenotypic sinonasal sarcoma features *PAX3* gene rearrangement [7].

The most common genetic alteration in glomangiopericytoma is a recurrent missense mutation within *CTNNB1* exon 3. This leads to the overexpression of cyclin D1 and oncogenic activation [1]. Recently, Hong et al. reported a GPC case with both *CTNNB1* and *PIK3CA* mutations. They also noted low tumor mutation burden in their case [8]. Additionally, DNA methylation analysis showed that glomangiopericytomas cluster close to normal sinus mucosa [9]. The tumor is indolent, but recurrence occurs in 20% of cases due to incomplete excision. Therefore, prolonged follow-up is recommended. Features that predict aggressive behavior include tumor size over 5 cm, severe nuclear pleomorphism, more than four mitoses per 2 mm^2^, skull base extension, and tumor necrosis [1]. Glomangiopericytoma is managed with endoscopic nasal surgery. In some cases, preoperative ligation is performed to control bleeding [10].

## Conclusions

Sinonasal GPC diagnosis can be challenging because it is a rare neoplasm with no specific clinical or radiological findings, with a wide range of histopathological differential diagnoses. Microscopic and immunohistochemical studies are crucial in the evaluation of a spindle cell neoplasm, especially with the overlap between the different entities. Long-term follow-up is recommended due to recurrence risk.
